# Regulation of Delta-Aminolevulinic Acid Dehydratase by Krüppel-Like Factor 1

**DOI:** 10.1371/journal.pone.0046482

**Published:** 2012-10-03

**Authors:** Aurelie D. Desgardin, Tatiana Abramova, Tolulope O. Rosanwo, Sreedharan Kartha, Eun-Hee Shim, Stephen M. Jane, John M. Cunningham

**Affiliations:** 1 Section of Hematology/Oncology and Stem Cell Transplantation, Department of Pediatrics, University of Chicago, Chicago, Illinois, United States of America; 2 Committee on Development, Regeneration, and Stem Cell Biology, University of Chicago, Chicago, Illinois, United States of America; 3 Australian Centre for Blood Diseases, Monash University Department of Clinical Hematology, Alfred Hospital, Melbourne, Australia; The National Institute of Diabetes and Digestive and Kidney Diseases, United States of America

## Abstract

Krüppel-like factor 1(KLF1) is a hematopoietic-specific zinc finger transcription factor essential for erythroid gene expression. In concert with the transacting factor GATA1, KLF1 modulates the coordinate expression of the genes encoding the multi-enzyme heme biosynthetic pathway during erythroid differentiation. To explore the mechanisms underpinning KLF1 action at the gene loci regulating the first 3 steps in this process, we have exploited the K1-ERp erythroid cell line, in which KLF1 translocates rapidly to the nucleus in response to treatment with 4-OH-Tamoxifen (4-OHT). KLF1 acts as a differentiation-independent transcriptional co-regulator of delta-aminolevulinic acid dehydratase (*Alad*), but not 5-aminolevulinate synthase gene (*Alas2*) or porphobilinogen deaminase (*Pbgd*). Similar to its role at the β-globin promoter, KLF1 induces factor recruitment and chromatin changes at the *Alad1b* promoter in a temporally-specific manner. In contrast to these changes, we observed a distinct mechanism of histone eviction at the *Alad1b* promoter. Furthermore, KLF1-dependent events were not modulated by GATA1 factor promoter co-occupancy alone. These results not only enhance our understanding of erythroid-specific modulation of heme biosynthetic regulation by KLF1, but provide a model that will facilitate the elucidation of novel KLF1-dependent events at erythroid gene loci that are independent of GATA1 activity.

## Introduction

Erythropoietic differentiation requires the orchestrated expression of tissue-specific and constitutive genes. Genetic analysis has demonstrated that a small group of hematopoietic-specific transacting factors are essential for effective erythroid-specific gene transcription [1], [2], [3], [4]. Krüppel like factor 1 (KLF1), also known as EKLF, was described initially as a β-globin promoter binding factor [5]. Further characterization of events at the *β-globin* gene cluster revealed key roles for KLF1 in modulating promoter chromatin architecture, recruitment of the upstream locus control region enhancer, the *γ*- to *β-globin* isotype switch, and *β*-gene transcriptional activation [6], [7], [8], [9], [10].

KLF1 is essential for definitive murine erythropoiesis, KLF1-null mice dying of anemia at E15.5 of gestation [11], [12]. Extensive studies have correlated expression of KLF1 and that of numerous erythroid-restricted genes required for progenitor proliferation and differentiation, including cell cycle regulators, synthetic enzymes, and components of the unique membrane and cytoskeletal structures of the mature erythrocyte [13], [14], [15], [16], [17]. Structure-function studies of naturally occurring and experimental KLF1 mutants reveal variable effects on these KLF1-dependent non-globin promoters, that differ significantly from those observed at the *β-globin* gene [18], [19], [20], [21], [22]. Together, these observations suggest that studies of KLF1 action at non-globin genes may delineate context-specific mechanism(s) of action of this factor, and provide insights into key targets required for effective erythropoiesis.

The heme biosynthesis pathway is critical for the development of the appropriate oxygen-carrying capacity of the erythrocyte. Coordinate expression of gene loci expressing 8 enzymes is required for effective heme synthesis. Fetal liver erythroblasts derived from KLF1-null mice demonstrate greatly diminished, but not absent, mRNA levels of the first three enzymes of the pathway [15], [16]. These enzymes catalyze the formation of 5-aminolevulinic acid (ALA) (ALA synthetase (ALAS2)), and the subsequent generation of porphyrin intermediates (ALA-dehydatase (ALAD) and porphobilinogen deaminase (PBGD)).

Studies to address the precise role of KLF1 in modulating transcription at gene loci outside the *β-globin* gene cluster have been confounded by the variable influence of differentiation status on erythroid-specific gene transcription. In contrast, a clear understanding of the essential role of the ‘master’ regulator GATA1 in erythroid specification, differentiation and tissue-specific gene expression has been facilitated by the use of inducible cell lines derived from GATA1 null erythroblasts [23], [24], [25]. To address the role of KLF1 in the regulation of heme biosynthesis, and its potential synergy with GATA1, we have taken advantage of a KLF1-inducible erythroid progenitor model to characterize the earliest events necessary for transcriptional activation [25]. Our studies demonstrate that KLF1 binds to the erythroid promoter of the *Alad* gene *in vitro* and *in vivo*. Unlike *Alas2* or *Pbgd*, KLF1 induced a rapid and substantial increase in *Alad* mRNA transcripts, enhancing the transcriptional rate being independent of cell differentiation. Our studies allow the separation of the role of GATA1 from KLF1 *in vivo*, characterizing key molecular events triggered specifically by KLF1 binding.

## Results

### *Alad* is Induced Specifically by KLF1 in K1-ERp Cells

Examination of global gene expression in KLF1-null murine fetal liver erythroblasts revealed that mRNA of the first three enzymes of the heme biosynthesis pathway was underrepresented, consistent with KLF1-regulated expression of these genes [13], [16]. *Alad* gene transcription in erythroid cells is regulated by a tissue-specific promoter (*Alad1b*) harboring consensus-binding sequences for GATA1 as well as a conserved CACC element [26], [27]. Similar motifs exist in the *Alas-2* and *Pbgd* gene promoters [11], [28], [29]. ChIP-Seq analysis using a KLF-specific antibody demonstrated an enrichment of these promoters in fetal erythroid progenitor cells [13]. Interestingly, the binding to *Alas-2* and *Pbgd* promoters was weaker, suggesting that KLF1 may play a more prominent role in the regulation of *Alad1b*-mediated transcription.

**Figure 1:**
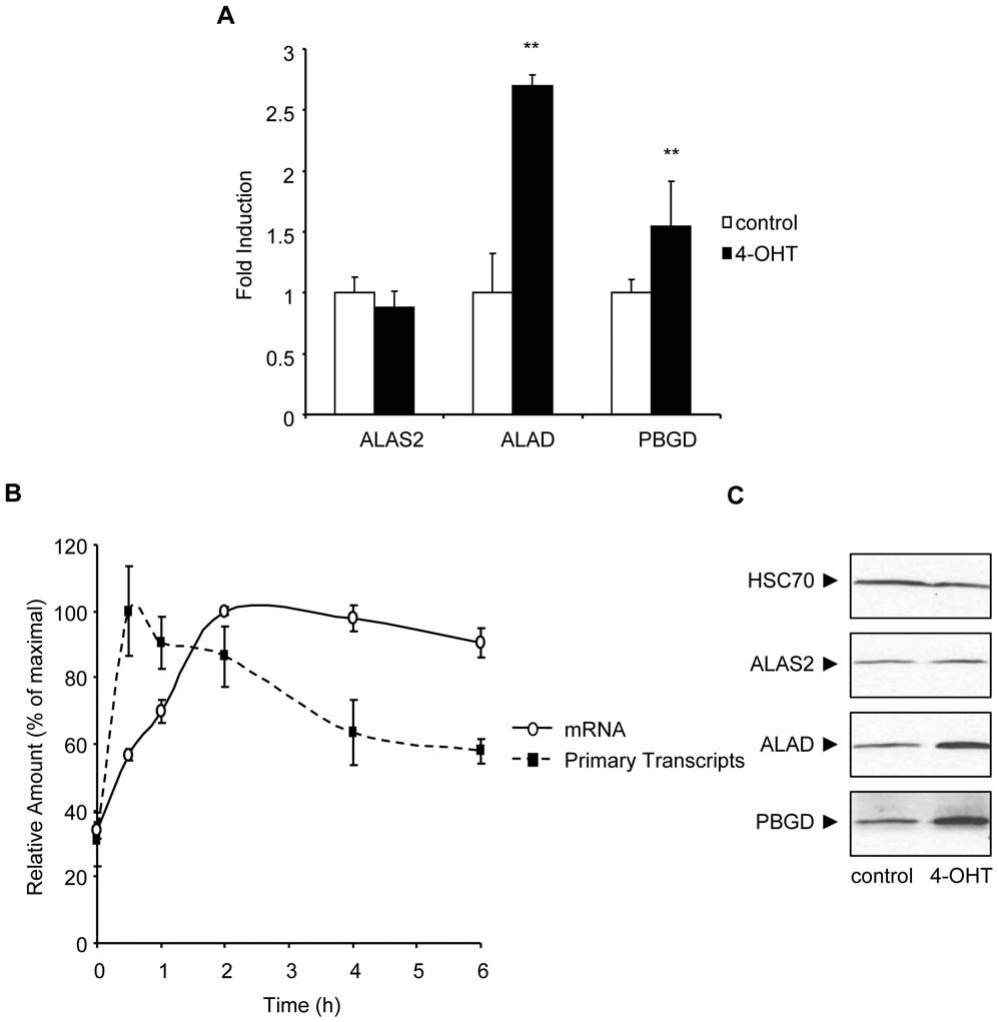
A significant change in *Alad* transcription with induction of KLF1 expression. (**A**) Relative mRNA levels of *Alas2*, *Alad* and *Pbgd* in K1-ERp cells treated with vehicle control or 4-OHT for 6 h, as determined by Q-RT-PCR. Represented mRNA levels were corrected to *Hprt* mRNA levels. (**B**) Relative RNA levels of *Alad* in K1-ERp cells corrected to *Hprt* mRNA levels, as determined by Q-RT-PCR. RNA was isolated at 0, 0.5,1,2,4 and 6 h after treatment of the cells with 4-OHT. (**C**) Western blot analysis of ALAS2, ALAD, PBGD proteins in K1-ERp cells treated with vehicle control or 4-OHT for 6 h. Equivalency of protein loading was verified with immunoblotting with HSC70-specific antisera *p value≤0.05. **p value ≤0.01 by Student’s *t*-test. Data shown represents the average of at least 3 independent experiments (mean ± SEM).

To explore these differences, we evaluated KLF1-dependent *Alad*, *Alas-2* and *Pbgd* transcription in K1-ERp cells. This erythroid model, derived from KLF1-null fetal erythroblasts, expresses a transgenic KLF1 cDNA, fused in frame to sequences encoding a hemagglutinin (HA) epitope, and regulated by an estrogen receptor-dependent regulatory sequence [30]. Exposure of KLF1-transduced cells to tamoxifen (4-OHT) results in rapid translocation of KLF1 to the nucleus. Associated with this change, we observed an induction of *Alad1b* mRNA levels over a 6 h time period as measured by semi-quantitative real-time reverse transcription PCR (Q-RT-PCR) (Fig. 1A). This increase in transcript levels phenocopies that observed between KLF1-null and wild type primary murine erythroblasts [15], [16]. Interestingly, we noted no significant change in *Alad1b* mRNA levels between K1-ERp cells and the parental KLF1 null cell line (Fig. S1), consistent with the idea that a functional transgenic KLF1 protein was dependent on 4-OHT exposure. In contrast, we observed no increase in *Alas2*, and only a minor increase in *Pbgd* mRNA levels during the same induction period, supporting previous reports suggesting that KLF1 plays a non-transcriptional role in the expression of these genes [11], [16]. Expression of other heme biosynthesis enzyme encoding genes did not change with 4-OHT induction of K1-ERp cells (data not shown).

To determine whether the increase in *Alad* mRNA levels is a result of changes in transcription efficiency, or of mRNA stability, we monitored the induction of primary RNA transcripts from the *Alad* locus. We observed a rapid and significant increase in *Alad* primary transcript levels upon 4-OHT treatment of K1-ERp cells (Fig. 1B). Interestingly, the transcriptional rate of *Alad* appeared to be modulated by additional mechanisms to maintain stable mRNA levels, as the decrease in *Alad* primary transcripts correlated with a stabilization of *Alad* mRNA levels. The increase in mRNA corresponded to a comparable increase in ALAD protein levels (Fig. 1C). Finally, a significant increase in the amount of PBGD protein level was also detected, despite the minimal increase in mRNA transcripts.

### KLF1 Binds and Activates the Erythroid Specific Promoter of *Alad in vitro* and *in vivo*

**Figure 2:**
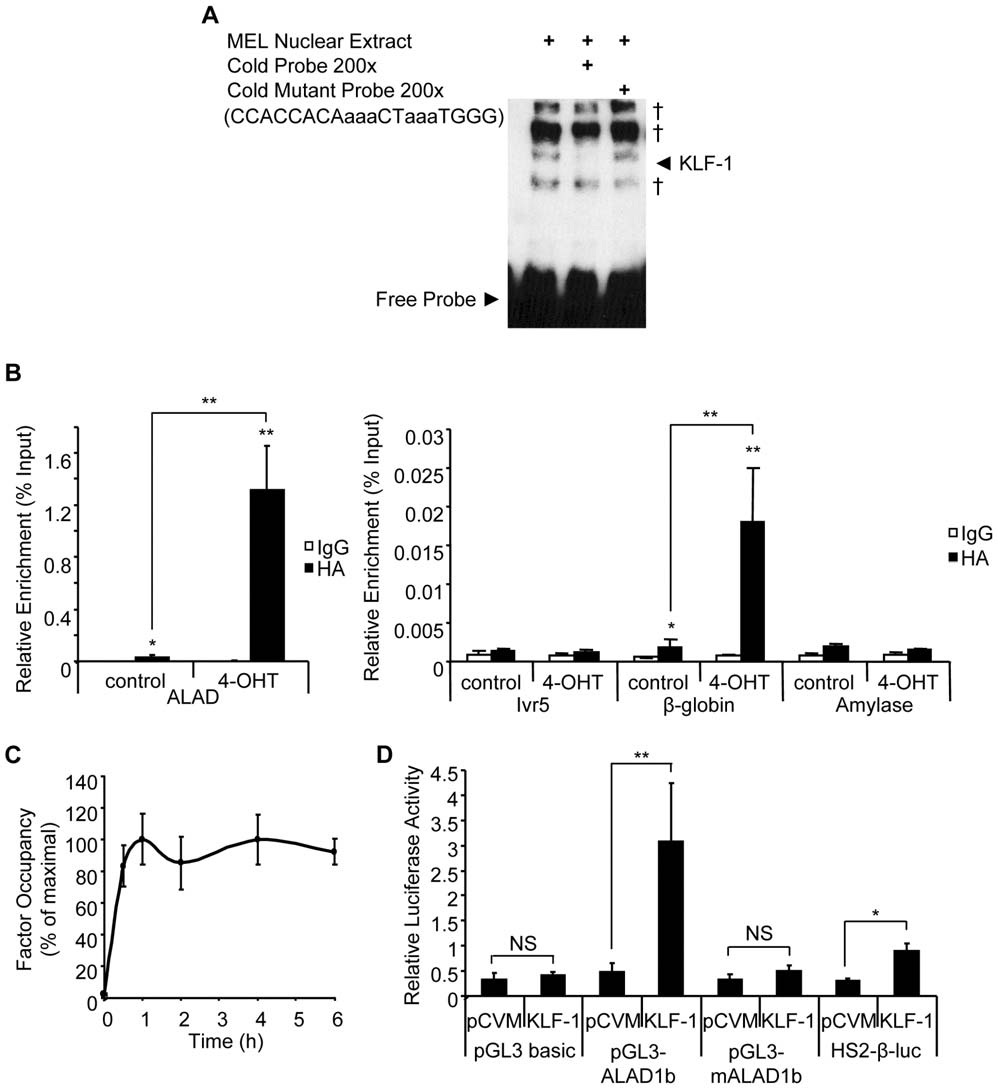
KLF1 binds at the *Alad1b* promoter in K1-ERp cells. (**A**) EMSA using a probe specific for the CACC box of the *Alad1b* promoter. Binding specificity was confirmed with the use of a mutated probe. † represents non-specific signal. (B + C) ChIP performed chromatin derived from K1-ERp cells treated with either vehicle control or 4-OHT. An antibody specific to HA epitope tag was used. Target DNA enrichment relative to input was determined by Q-PCR. (**B**) Sequences used were *Alad1b*-specific encoding the CACC element, β-globin and amylase promoter-specific as positive and negative controls respectively. Ivr5 primers, located approximately 1 kb upstream of the *β-globin* promoter served as a control for sonication conditions. (**C**) Kinetic analysis of KLF1 occupancy at the *Alad1b* promoter relative to maximum detected occupancy as determined by ChIP performed on K1-ERp cells harvested at 0, 0.5, 1,2, 4 and 6 h into 4-OHT treatment. (**D**) Dual luciferase reporter assay performed in K562 cells in the presence of vector only or KLF1 expression vector. Mean relative luciferase levels were corrected to Renilla luciferase levels. HS2-*β*-luc construct contains the *β-major* CACC element and was used as a positive control. pGL3-m*Alad1b* corresponds to the *Alad1b* promoter sequence bearing mutations within the CACC element. *p value≤0.05. **p value ≤0.01 by Student’s *t*-test. Data shown represents the average of at least 3 independent experiments (mean ± SEM).

To demonstrate binding of KLF1 to the CACC element of the *Alad* promoter, we performed electrophoretic mobility shift assays, using nuclear extracts from MEL cells. The importance of this study is highlighted by previous observations defining both DNA consensus binding sites similar to that observed at the murine erythroid-specific *Alad* promoter (-97 relative to the transcriptional start site) and highly variant motifs as seen at the *Ahsp* erythroid gene locus [26]. As shown in Fig. 2A, a specific CACC binding complex is visualized with the wild type *Alad* probe. Binding activity is lost with co-incubation of excess unlabeled probe. In contrast, a mutated CACC element probe failed to influence complex binding.

To ascertain whether KLF1 is recruited to the *Alad1b* promoter *in vivo*, we performed KLF1-specific ChIP assays in K1-ERp cells. Enrichment of the *Alad1b* promoter was detected in 4-OHT treated KLF1 transgenic cells only (Fig. 2B). As relevant controls, KLF1 was also detected at the β-globin promoter, but did not occupy the non-erythroid amylase gene promoter, nor a region located approximately 1 kb upstream of the *β-globin* promoter. As the rate of transcription of the *Alad1b* gene gradually decreased over time, we assessed the stability of KLF1 binding to the *Alad1b* promoter. Examination of the kinetics of KLF1 recruitment to the *Alad1b* promoter at multiple time points post 4-OHT exposure revealed maximal binding levels within the first 30 min of induction, correlating with the measured kinetic response in *Alad1b* transcription (Fig. 2C). KLF1 occupancy remained maximal for the duration of the analysis. These findings suggest that the decrease in the transcriptional rate of *Alad1b* observed previously is not a consequence of a decrease in KLF1 promoter occupancy.

To assess KLF1’s ability to transactivate the *Alad* erythroid promoter, we employed a dual-luciferase reporter assay. The *Alad* promoter (−1 to −270) was cloned into pGL3-Basic vector and co-transfected into K562 cells (which express low endogenous levels of KLF1), with a renilla luciferase expression construct and either a KLF1 expression vector (pCMV-KLF1) or a vector control (pCMV). The HS2-*β*-Luc construct was used as a positive control for KLF1-dependent transactivation [31]. HS2-*β*-Luc and the *Alad1b* promoter were activated only in the presence of KLF1 (Fig. 2D). Importantly, KLF1 did not activate the minimal pGL3Basic regulatory sequences, nor an *Alad1b* promoter with CACC mutations, indicating that transactivation requires an intact binding motif. Cell cycle monitoring via *p18* expression analysis indicated that KLF1 over-expression did not induce the differentiation of K562 cells (Fig. S2) [32], [33], [34]. This data suggests that KLF1 is able to modulate transactivation of the *Alad1b* promoter independent of erythroid differentiation.

### KLF1 Binding to the ALAD Promoter is Associated with the Mobilization of Erythroid Transacting Factors

**Figure 3:**
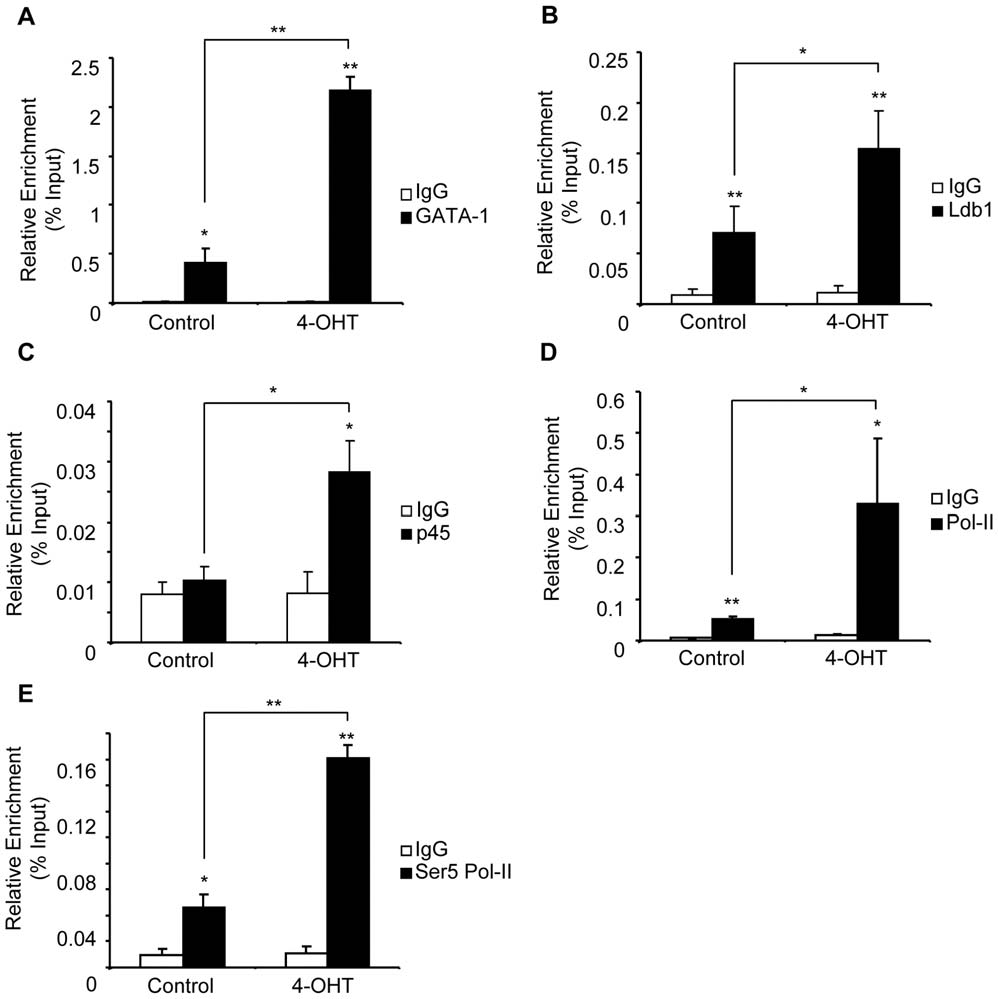
KLF1 recruits specific and general transcription factors to the promoter of *Alad1b*. ChIP performed on chromatin derived from K1-ERp cells treated with either vehicle control or 4-OHT. Antibodies specific to (**A**) GATA1, (**B**) Ldb1, (**C**) p45NF-E2, (**D**) RNA Pol-II and (**E**) Ser5 RNA Pol-II were utilized. Target DNA enrichment relative to input was determined by Q-PCR using *Alad1b* promoter primers. *p value≤0.05. **p value ≤0.01 by Student’s *t*-test. Data shown represents the average of at least 3 independent experiments (mean ± SEM).

Synergistic interactions between KLF1 and GATA1, and between GATA1 and the hematopoietic transacting factor SCL/TAL-1 multiprotein complex, have been reported to be essential for maximal erythroid gene transcription, prompting us to evaluate their potential relationship in the transcriptional regulation of *Alad* [35], [36], [37], [38], [39]. The *Alad1b* promoter exhibits multiple GATA1 binding sites, as well as an E-box at −227 [26]. SCL/TAL-1 is known to bind E-box elements associated with GATA1 binding sites in erythroid cells [38], [39], [40]. We hypothesized that GATA1 and SCL/TAL-1 modulate *Alad1b* expression in erythroid cells. To test our model, we examined *Alad1b* promoter occupancy by GATA1 and Ldb-1, the latter factor being a key subunit of the SCL/TAL-1 complex. In the absence of KLF1, both factors were identified at the promoter (Fig. 3A and B respectively). A differential enhancement of factor binding occurred with KLF1 promoter occupancy, with a more significant change in GATA1 binding when compared to Ldb-1.

In addition to GATA1, SCL/TAL-1 and KLF1, the NF-E2 protein complex is required to promote *β-globin* gene transcription and coordinates expression of genes of the heme biosynthesis pathway [41], [42], [43], [44], [45], [46], [47]. Although a consensus binding site for the p45NF-E2 complex (TGA(G/C)TCA-3′/3′-ACT(C/G)AGT) is not present in the erythroid *Alad* promoter, NF-E2 has been detected at other KLF1-target genes in the absence of this sequence [48], [49], [50]. To examine p45NF-E2 occupancy of the *Alad1b* promoter in 4-OHT treated K1-ERp cells we used a p45NF-E2 specific antibody in ChIP analysis. We demonstrated that p45NF-E2 was recruited to the promoter in the presence of KLF1 (Fig. 3C). However, unlike GATA1 and Ldb-1, p45NF-E2 was not detected prior to KLF1 binding. Together, our data suggests that GATA1 and SCL/TAL-1 complex are sufficient to drive low levels of transcription of the *Alad* gene. However, maximal occupancy of the GATA1 complex at the *Alad1b* promoter, and recruitment of p45NF-E2 is dependent on KLF1.

One possible explanation for the changes in factor occupancy is that KLF1 induces significant transacting factor transcription during the 6 hour observation period. To test this possibility, we examined mRNA levels of *Gata-1*, *Ldb-1*, and *p45Nf-e2* before and after 4-OHT exposure (Fig. S3). We observed a decrease in *Gata-1* expression, no change in *p45Nf-e2* mRNA, and a small but significant increase in *Ldb-1* mature transcripts. These findings suggest a complex interplay between the effects of KLF1 on factor recruitment, and changes in cofactor expression.

### KLF1 Binding Enhances Recruitment of the RNA Polymerase II (RNA Pol-II) Complex

The low levels of *Alad* transcripts detectable in KLF1-null cells suggest that KLF1 is not essential for basal *Alad* gene transcription. This observation contrasts with the absolute requirement of KLF1 for the transcriptional activation of the *β-globin*, *Ahsp* and *Dematin* genes [11], [15], [31], [51]. To evaluate the role of KLF1 in the recruitment of RNA Pol-II transcriptional complex to the *Alad1b* promoter, we performed ChIP assays in K1-ERp cells in the presence and absence of 4-OHT. Binding of RNA Pol-II was demonstrable at the promoter in control cells, consistent with active transcription (Fig. 3D). RNA Pol-II occupancy increased with 4-OHT treatment.

The activation state of RNA Pol-II influences the rate of transcription. Active RNA Pol-II is phosphorylated at the Ser5 residue of its CTD [52]. To evaluate the impact of KLF1 recruitment on the phosphorylation status of RNA Pol-II, we monitored Ser5 Pol-II occupancy at the *Alad1b* promoter in the absence or presence of KLF1. As anticipated, our results revealed occupancy of the activated form of RNA Pol-II at the *Alad1b* promoter in control cells. The increase in Ser5 Pol-II observed at the *Alad1b* promoter upon 4-OHT treatment (Fig. 3E) was comparable to the measured increase in total RNA Pol-II. Therefore, our results suggest that KLF1 binding to the ALAD1b promoter induces the enhanced recruitment of RNA Pol-II to the promoter but not its activation.

### KLF1 Induces Alterations in Chromatin Structure at the ALAD1b Promoter

**Figure 4:**
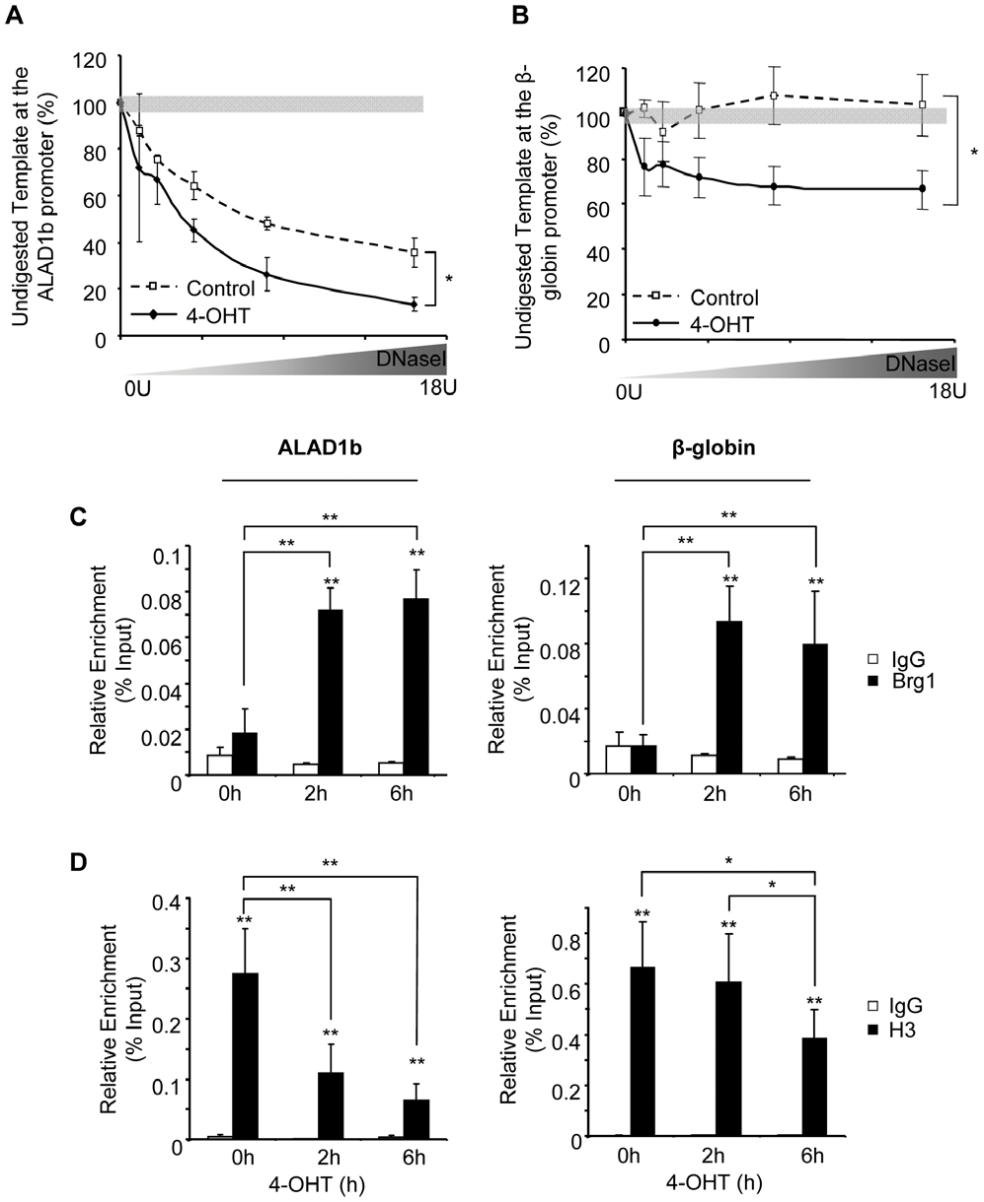
KLF1 enhances DNaseI hypersensitivity site formation at the *Alad1b* promoter. (**A+B**) DNaseI hypersensitivity assay in K1-ERp cells treated with vehicle control or 4-OHT at the *Alad1b* and *β-globin* promoters respectively. (**C+D**) ChIP performed on chromatin derived from K1-ERp cells treated with either vehicle control or 4-OHT. Antibodies were specific to (C) Brg1 or (D) histone H3. Target DNA enrichment relative to input was determined by Q-PCR using *Alad1b* or *β-globin* promoter primers. *p value≤0.05. **p value ≤0.01 by Student’s *t*-test. Data shown represents the average of at least 3 independent experiments (mean ± SEM).

KLF1 binding at various erythroid-specific regulatory elements induces a change in chromatin architecture, as measured by DNaseI hypersensitivity [31], [53], [54]. Thus, KLF1-mediated chromatin remodeling could account for the observed enhancement of *Alad* transcription, by increasing promoter accessibility to additional factors. To address this, we measured the DNaseI hypersensitivity of the *Alad1b* promoter in K1-ERp cells. As shown in Fig. 4, the *Alad1b* promoter was sensitive to DNaseI digestion in the absence of KLF1, contrasting with a lack of DNaseI sensitivity at the KLF1-dependent control *β-globin* promoter (A and B respectively). This particular observation further validates the specificity of the system as chromatin remodeling at the β-globin promoter is extremely sensitive to KLF1 [53], [54]. In contrast, increased DNaseI sensitivity was observed at the *Alad1b* promoter 6hrs after exposure to 4-OHT compared to vehicle control cells.

The ability of KLF1 to remodel the chromatin of the *β-globin* locus is associated with recruitment of a SWI/SNF-encoding multiprotein complex [8]. ChIP analysis in K1-ERp cells revealed that Brg1, the ATPase subunit of this complex was recruited to the *β-globin* promoter as well as the *Alad1b* promoter in the presence of KLF1 only (Fig. 4C). This data suggests that GATA1 together with the SCL/TAL-1 complex are not sufficient to recruit Brg1, and that Brg1 binding is not necessary for basal *Alad1b* promoter activity.

To determine whether the transcriptional status of genes influences KLF1-directed chromatin remodeling, we evaluated the nucleosomal density at the *Alad1b* and *β-globin* promoters. Using histone H3 as a marker of nucleosomes, we determined by ChIP analysis that histone H3 density remains unchanged during the first 2 h of 4-OHT treatment at the *β-globin* promoter, eventually decreasing, probably as a consequence of *de novo* transcription and multiple passages of elongating RNA Pol-II (Fig. 4D). In contrast, the *Alad1d* promoter displayed a marked decrease in bound histone H3 within the first 2 h of 4-OHT treatment. As *Alad1b* is transcribed prior to KLF1 nuclear localization, the histone eviction cannot be attributed to the elongation process suggesting that KLF1 directly induces differential H3 loss.

### KLF1 Enhances Histone Modification at the ALAD1b Promoter

**Figure 5:**
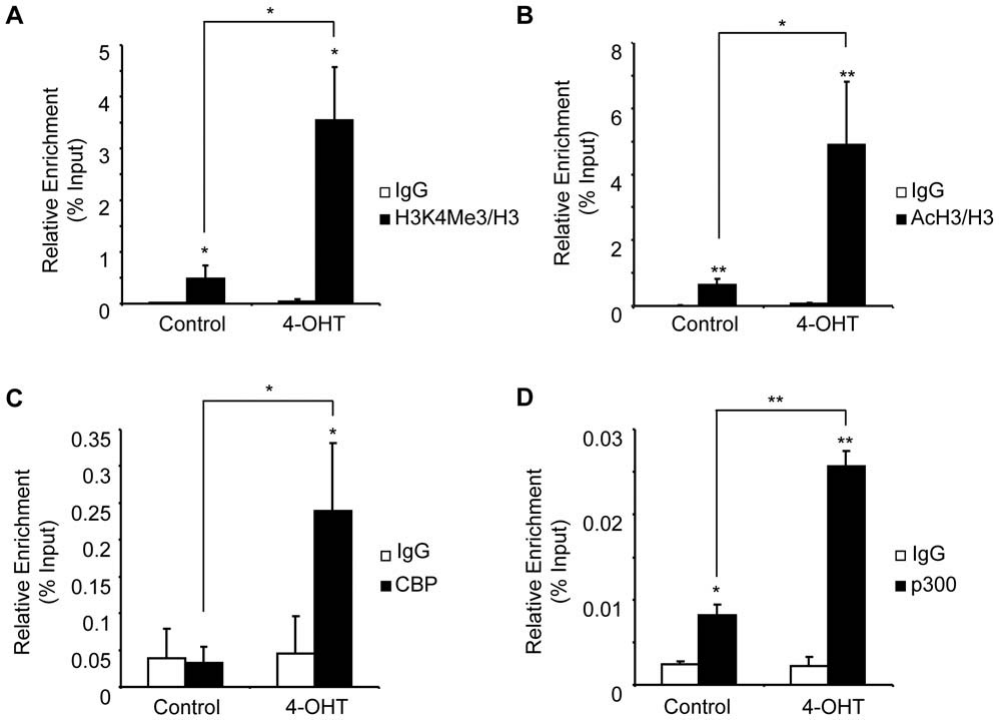
KLF1 enhances the deposition of histone modifications associated with active genes. ChIP performed on chromatin derived from K1-ERp cells treated with either vehicle control or 4-OHT. Antibodies were specific against (**A**) histone H3K4Me3, (**B**) acetylated histone H3, (**C**) CBP and (**D**) p300. Levels of H3K4Me3 and AcH3 were corrected to histone H3 occupancy. Target DNA enrichment relative to input was determined by Q-PCR using *Alad1b* promoter primers. *p value≤0.05. **p value ≤0.01 by Student’s *t*-test. Data shown represents the average of at least 3 independent experiments (mean ± SEM).

In addition to its chromatin remodeling potential, KLF1 induces covalent histone modifications associated with active genes (H3K4me3 and AcH3) at the *β-globin* locus [55], [56], [57], [58], [59], [60]. To gain insight into the mechanisms driving the deposition of these histone marks at the *Alad1b* gene, we determined the relationship between KLF1 binding, transcription, and histone mark deposition using our inducible cell system. We focused our efforts on the activation marks H3K4Me3 and AcH3 using ChIP analysis in the absence and presence of KLF1. No significant increase in the global levels of the histone modifications were detected after 6 hrs of exposure of K1-ERp cells to 4-OHT when compared to non-exposed cells (Fig. S4–A and B). However, correcting the measured levels of histone modification to the total levels of H3, we demonstrated that KLF1 binding at the active *Alad1b* promoter resulted in increased occupancy of H3K4Me3- and AcH3-modified histones at the promoter (Fig. 5A and B respectively).

The link between AcH3 and KLF1 binding suggested that KLF1 directly modulates the acetylation of histone H3 at the active *Alad1b* promoter. KLF1 is known to interact with three histone acetyltransferases (HATs) *in vitro*, namely CBP, p300 and P/CAF [61]. We examined the inter-relationship between KLF1 and CBP/p300 at the *Alad1b* promoter. As shown in Fig. 5C, CBP was not detected at the *Alad1b* promoter prior to KLF1 induction by 4-OHT. In contrast, promoter occupancy by p300 was substantial in control cells, and underwent further enhancement after 4-OHT treatment (Fig. 5D). Our data suggest that recruitment of CBP to the erythroid-specific *Alad* promoter is strictly dependent upon KLF1 binding, whereas p300 binding occurs in both KLF1-dependent and -independent settings. Our data suggests that KLF1-mediated histone acetylation arises at least in part via the recruitment of CBP, and enhanced binding of p300.

## Discussion

In KLF1-null fetal erythroblasts, heme biosynthesis enzyme levels are reduced globally, but not abolished [15], [16]. Previous studies have focused on the trans-acting factors responsible for the expression of two key enzymes, ALAS2 and PBGD. While *in vitro* studies suggested a regulatory role for KLF1 at these loci, *in vivo* studies failed to validate these findings [11], [13], [16]. In contrast, little is known about the regulation of expression of the second enzyme in the heme pathway, *δ-Aminolevulinate Dehydratase* (*Alad*). Taking advantage of our inducible cell-system, we show here that the *Alad1b* promoter is the first heme-regulatory sequence activated by KLF1 binding. Interestingly, over the 6 hours of 4-OHT exposure, levels of *Alas2* mRNA, a marker of ongoing erythroid differentiation, remain unchanged. In contrast, *Alad1b* transcription increases significantly. Thus, the *Alad1b* transcriptional response to KLF1 binding is both direct and terminal differentiation-independent.

Our studies suggest that *Alad* mRNA levels are influenced by complex regulatory signals in the first 6 hours after induction of KLF1. *Alad* primary transcript levels increased initially but were diminished 2 hrs after 4-OHT exposure. This decrease in transcriptional rate does not correlate with changes in KLF1 promoter occupancy or the levels of *Alad* mRNA. These observations suggest that activating and repressive mechanisms influence *Alad1b* transcription. We also observed a significant increase in PBGD protein levels that does not correlate with *Pgbd* mRNA transcripts, suggesting that KLF1 may play a role in the translational regulation of *Pbgd*. These observations emphasize the complexity of the regulatory mechanisms influencing heme biosynthesis.

*Alad* mRNA levels were induced 2.5 fold when compared to uninduced cells, suggesting that the *Alad* deficiency observed in KLF1-null erythrocytes contributes to the anemic phenotype. Support for this conclusion is provided by the hypochromic anemia phenotype in fish deficient in ALAD [62]. The essential function(s) of ALAD in mammalian erythropoiesis remain unclear. One clue may be its putative function in regulating proteosomal-mediated degradation during erythroblast differentiation [63], [64].

We show that a role of KLF is to augment rather than induce *Alad* gene transcription, a significant difference from its essential requirement for effective *β-globin* gene transcription. It is possible that KLF1 plays a similar role in chromatin remodeling, an early step in differentiation required at all erythroid loci [31], [65]. Unlike the *β-globin* gene, we observed only a modest increase in DNaseI sensitivity with KLF1 binding at the already hypersensitive *Alad1b* proximal regulatory sequence. In addition, and in contrast to the *β-globin* promoter, KLF1 binding to *Alad1b* induces a significant decrease in histone H3 promoter occupancy, consistent with the idea that KLF1 binding induces a local chromatin structural reconfiguration. This undescribed mechanism of KLF1 mediated chromatin remodeling, histone eviction, suggests more complex and context-specific mechanism(s) of KLF1 action than previously appreciated.

The SWI/SNF subunit Brg1 is a strong candidate for modulating KLF1-mediated histone eviction. Strong support for such a model comes from the essential role of KLF1:Brg1 interaction in nucleosomal phasing and induction of DNaseI hypersensitivity at the β-globin promoter [8], [30], [61], [66]. However, our studies suggest that the mechanism differs, *Alad1b* promoter DNaseI hypersensitivity predating KLF1-mediated recruitment of Brg1. Instead, these early events are associated with GATA1 and SCL/TAL-1 complex binding. Although the latter factors represent attractive candidates for regulating DNaseI hypersensitivity, it remains a possibility that their binding is not related causally to promoter accessibility. It will be important to explore the role of Brg1 thoroughly in histone eviction at the *Alad1b* promoter. Equally, it is possible that the role of Brg1 may vary completely, being required perhaps to enhance transcriptional initiation by recruitment of an upstream enhancer [67].

Changes in covalent histone modifications (AcH3, H3K4Me3) at the *Alad1b* promoter may also be involved in histone eviction. We corrected the level of histone modification for total histone H3, our results suggesting that histone eviction may be associated with replacement by a histone variant [68], [69], [70]. One replacement candidate is histone 3.3, a variant known to be incorporated at transcriptionally active genes. Recently, KLF1 has been implicated in this process, epitope tagged H3.3 being incorporated in a KLF1-dependent manner at the *β-globin* promoter upon MEL cell erythroid differentiation [60].

The K1-ERp cell system is particularly attractive for study as it allows us to distinguish between GATA1- versus KLF1-dependent during erythropoietic differentiation. For example, Ldb1 recruitment to the *Alad1b* promoter does not require KLF1. Similarly, GATA1 is present at the *Alad1b* promoter along with p300 and low levels of RNA Pol-II in the absence of KLF1. This is consistent with the idea that p300 but not CBP activity is required for differentiation-specific hematopoietic gene expression [71]. Interestingly, the mobilization of aforementioned factors to the *Alad1b* promoter is enhanced in the presence of KLF1. In a second step, recruitment of p45NF-E2, Brg1 and CBP occurs in a KLF1-dependent manner. This two-phase model of factor recruitment suggests that each factor combination plays a unique role in achieving maximal gene transcription.

Both HATs interact physically *in vitro* and *in vivo* with GATA1 and KLF1, yet they are not mobilized in a similar manner to the *Alad1b* promoter [61], [72]. One possible explanation for this difference is that p300 may subserve the role of priming the 3-dimensional structure of the promoter by histone and factor acetylation. This activity facilitates recruitment of KLF1 and the second group of factors. We suggest that CBP and NF-E2 are required to establish the appropriate milieu for recruitment of high levels of activated RNA-PolII, facilitating the relocation of the *Alad* locus to a subnuclear compartment rich in shared transcription factors [73]. Further studies using our cellular model will allow us to test this and alternate hypotheses.

In conclusion, we show here that maximal *Alad* transcription requires binding of KLF1 to its cognate promoter, occurring in the absence of ongoing erythroid differentiation. In contrast, *Alas2* and *Pbgd* transcription is KLF1- independent. We report a novel mode of chromatin remodeling triggered by KLF1 binding, with histone depletion. In addition, we have demonstrated a two-step mechanism of transcriptional activation of the *Alad* locus, with key transactivators requiring KLF1-binding. These observations provide further insight into the complex mechanisms of erythroid gene expression, and a platform for studies of the temporal events necessary for tissue-specific gene transcription.

## Materials and Methods

### Cell Culture

K1-ERp cells [15], [25] were maintained in the log phase of growth, between 0.2 and 1.5×10^6^ cells/ml, in D15puro (Dulbecco’s modified Eagle’s medium (DMEM) supplemented with 15% Fetal Calf Serum (FCS), 1% penicillin/Streptomycin and 1 µg/ml of puromycin (Invivogen)) at 37°C, 5% CO_2_. 2 units/ml of erythropoietin was added directly to the cells 3 days prior to cell harvest. The nuclear translocalization of KLF1 in K1-ERP cell was induced by the addition of 4-OH-Tamoxifen (4-OHT) to a final concentration of 100 nM. Murine Erythroleukemia cells (MEL) [74], J2e cells [25] and human erythroleukemia cells (K562, obtained from the ATCC repository) were maintained in DMEM supplemented with 10% FCS and 1% Penicillin/Streptomycin, at 37°C and 5% CO_2_.

### RNA Analysis

RNA was extracted using the Trizol method and cDNA was prepared using the Superscript II First Strand System (Invitrogen) following the manufacturer’s protocols. RNA (10 µg) was treated with Turbo DNase (2 µL) (Ambion) in a 50 µL reaction to remove genomic DNA contamination. DNase-treated RNA (2 µg) was used for first strand DNA synthesis. The resulting cDNA was diluted 1∶50 for mRNA analysis and 1∶2 for primary transcripts analysis.

2 µl of the cDNA product was subjected to semi-quantitative analysis using real-time PCR (Applied Biosystems Prism 7900) with the appropriate primer pairs. Products were quantified using SYBR green fluorescence in 20 µl reactions. Primer pairs were designed using the Clone Manager software to obtain PCR products of 50–150 bp. Primers used to detect *Alad1b* primary transcripts amplify the exon2-intron2 junction: Forward 5′ GCCTCCAACCTCATCTATCC-3′ and Reverse 5′- TCCCATTGCCTGTTCCAGTC-3′. Primers used to measure mRNA were as followed: *Alas2* Forward 5′-CTTTG ATCTCAGGACTGCTG-3′ and Reverse 5′-AACAGCTGCGGTGCAAAGTA-3′, *Alad1b* Forward 5′-CACCTATGCTTAAGGAGCCA-3′ and reverse 5′-CAGAAGCACACAGGAAAGCA-3′, *Hprt* Forward 5′- GCAGTACAGCCCCAAAA TGG-3′ and Reverse 5′-AACAAAGTCTGGCCTGTATCCAA-3′, *Pbgd* Forward 5′- TACTTCTGGCTTCCAAGTGC-3′ and Reverse 5′-CAAGGTGAGGCATATCTTCC-3′.

### Protein Analysis

Whole cell extracts from ethanol treated and 4-OHT treated K1-ERp cells were obtained by lysis in 1% NP-40 lysis buffer. 50 µg of lysate was diluted with 6X SDS-loading buffer (1.2% SDS, 0.6% bromophenol blue, 47% glycerol, 60 mM Tris-Cl pH6.8, 0.6 M DTT ), denatured by boiling for 3 minutes and resolved on a 10% or 12% SDS-PAGE gel. Proteins were then transferred to a nitrocellulose membrane by wet transfer procedure. Primary antibody incubation with relevant antibodies was carried out overnight at 4°C. Secondary antibody incubation was carried out at a dilution of 1: 3,000 of anti-rabbit or 1: 10,000 anti-goat horseradish peroxidase (HRP) conjugated antibodies in 5% milk in 1X PBS, 0.1% Tween-20 for 1 hr. Peroxidase activity was detected by enhanced chemiluminescence (ECL) using detection reagents from Pierce followed by exposure to X-ray films.

### Electrophoretic Mobility Shift Assay (EMSA)

Nuclear extracts from MEL cells were prepared using a modified Dignam’s protocol [75]. The EMSA was carried out using the LightShift® Chemiluminescent EMSA kit (Pierce), following the manufacturer’s instructions. Double-stranded oligonucleotide –CCCACCCAGGGGTGTGGTGG- (CACC probe) was used as the probe for EMSA. The 20 µl EMSA reactions contained 5 µg of MEL cell nuclear extract, 2 µl of 10X binding buffer provided in the kit, 2.5% Glycerol, 0.05% NP-40, 5 mM MgCl_2,_ 20 fmol of Biotin-End labeled probe and dH_2_O to a final volume of 20 µl. A separate control reaction was prepared omitting the nuclear extract. After 20 min incubation at room temperature, 5 µl of 5X loading buffer was added and samples were loaded onto a 5% polyacrylamide gel. Samples were separated by electrophoresis in 0.5X TBE buffer. To assess binding specificity, a second control was prepared adding a 200X molar excess of unlabeled CACC proximal probe for competition. A third control was prepared adding a 200X molar excess of the non-specific mutated CACC proximal probe –CCCATTTAGTTTTGTGGTGG-. Mutated nucleotides are underlined.

### Chromatin Immunoprecipitation (ChIP)

ChIP assays were performed as previously described, with slight modifications [76], [77]. Briefly, protein-DNA and protein-protein crosslinking from ethanol-treated or 4-OHT treated K1-ERp cells was achieved by the addition of formaldehyde to a final concentration of 1% followed by a ten minute incubation at room temperature. The reaction was quenched by the addition of glycine to a final concentration of 0.125 M for 5 min at room temperature. Cells were collected by centrifugation at 1,000 rpm for 5 min at 4°C and washed twice in cold PBS. 1×10^7^ cells per immunoprecipitation condition were resuspended in cold cell lysis buffer (10 mM Tris-HCl pH8.0, 10 mM NaCl, and 0.2% NP-40) supplemented with protease inhibitors and incubated on ice for 10 min. Nuclei were then resuspended in 300 µl of nuclear lysis buffer (50 mM Tris-HCl pH8.0, 10 mM EDTA, 1% SDS) supplemented with protease inhibitors and incubated for 10 min at 4°C. Chromatin was sheared by sonication using the Bioruptor sonicator (Diagenode) to achieve an average DNA fragment size of 500–2000 bp. Soluble chromatin was collected and diluted 1 to 4 with cold immunoprecipitation buffer (20 mM Tris-HCl pH8.0, 2 mM EDTA, 150 mM NaCl, 1% Triton-X100, and 0.1% SDS) supplemented with protease inhibitors. Samples were pre-cleared by adding 50 µl of salmon sperm DNA/protein A/G agarose beads (Millipore) and 5 µl/ml of rabbit serum. Antibody (2–5 µg), or appropriate normal IgG for control, were added to the soluble chromatin overnight at 4°C. Immune complexes were adsorbed to 70 µl of salmon sperm DNA/protein A/G agarose beads (Millipore) for 2 hrs at 4°C. Beads were washed twice in cold wash buffer 1 (20 mM Tris-HCl pH8.0, 2 mM EDTA, 50 mM NaCl, 1% Triton X-100, and 0.1% SDS) followed by 1 wash with cold wash buffer 2 (10 mM Tris-HCl pH8.0, 1 mM EDTA, 250 mM LiCl_2,_ 1% NP-40, and 1% Deoxycholic acid) and 2 washes with Tris-EDTA buffer. Protein-bound DNA was eluted from the beads by 2 sequential incubations of 15 min each with elution buffer (50 mM NaHCO_3_, 1%SDS). Crosslinks from immunoprecipitation samples and inputs were reversed by the addition of 1 µl of 20 mg/ml RNaseA (Ambion) and overnight incubation at 65°C. DNA purification was carried out using a Qiaquick PCR purification kit (QIAGEN) following manufacturer’s instructions. Analysis of the purified DNA was achieved using real-time PCR (Applied Biosystems Prism 7900) with the appropriate primer pairs. Products were quantified using SYBR green fluorescence in relation to a standard curve generated from serial dilutions of an input sample. PCR efficiency and specificity was determined by analysis of the standard curve and the dissociation curve respectively. Primer pairs were designed using CloneManager software to obtain PCR products of 50–150 bp. Primer sequences are presented in Table 1.

**Table 1:** Primers used for Chromatin Immunoprecipitation studies.

Region of amplification	Forward (5′-3′)	Reference
	Reverse (5′-3′)	
***Alad1b*** ** promoter**	ctcttgtgtcctgtgaagag	Self designed
	caagcagctcagggcccaccttatc	
***Alad*** ** upstream (−1.3** **kb)**	cctgtgcctcatacagtaac	Self designed
	gtttggcccagaaacagttg	
***Amylase*** ** promoter**	ctccttgtacgggttggt	[81]
	aatgatgtgcacagctgaa	
***β-major*** ** promoter**	gggagaaatatgcttgtcatc	[81]
	caactgatcctacctcacctt	
***Ivr5***	gtatgctcaattcaaatgtaccttattttaa	[77]
	ttacctctttatttcacttttacacatagctaa	

### Reporter Assays

Promoter-reporter assays were conducted using the Dual-Luciferase Reporter assay system from Promega following manufacturer’s instructions. 0.5×10^6^ K562 cells were co-transfected with pTk-Rn (0.04 µg) and pGL3-basic or pGL3-basic containing ALAD1b promoter sequence (0.5 µg) with either pCMV or pCMV-KLF1 (1 µg). Firefly luciferase activity was assayed 24 hr after transfection and corrected to Renilla luciferase activity. An HS2-β-Luc construct was used as a positive control for a KLF1 dependent promoter sequence [31], [78].

### DNase I Sensitivity Assays

DNaseI sensitivity assays were performed essentially as described with slight modifications [79]. K1-ERp cells (1.2×10^7^) were washed once with cold PBS and resuspended in 14 ml RSB (10 mM Tris HCl (pH 7.5), 3 mM MgCl_2_, and 10 mM NaCl). Cells were lysed in the presence of 0.25% (v/v) NP-40, and nuclei were collected and resuspended in 1.5 mL RSB. The nuclei mixture (200 µL) was added to 30 µL of an enzymatic mix containing 0–18 units of DNaseI (ROCHE). DNaseI digestion was carried out at 37°C for 10 minutes and terminated by the addition of an equal volume of stop buffer (600 mM NaCl, 1% (w/v) SDS, 20 mM Tris-HCl (pH 7.5), and 10 mM EDTA). The samples were subjected to proteinase K (500 µg/mL) treatment overnight at 55°C. DNA was recovered by extraction with phenol/chloroform followed be ethanol precipitation. DNA analysis was performed using real-time PCR with SYBR green fluorescence detection. Quantification of the undigested template was achieved in relation to a standard curve generated from serial dilutions of undigested genomic DNA. Results were normalized to those obtained for a previously determined DNaseI insensitive gene, NF-M [80]. PCR efficiency and specificity was determined by the analysis of the standard and dissociation curves respectively. Control primers used for the assay are as followed: *Nf-m* forward: 5′- GCTGGGTGATGCTTACGACC-3′ and reverse: 5′- GCGGCATTTGAACCCTCTT-3′ [80]. Additional primer pairs used for the assay were identical to those used for the ChIP studies.

### Antibodies

Antibodies used for western blotting are as follows: anti-HSC70 (K-19) at a dilution of 1∶2000, anti-ALAS2 (D-4), anti-ALAD (A-7) and anti-PBGD (A-16) at a dilution of 1∶500 (SantaCruz Biotech). Antibodies used for chromatin immunoprecipitation studies are as follows: anti-HA (Y11), anti-GATA1 (N16), anti-Clim2 (N18), anti-NFE2 (C19), anti-CBP (A22), anti-p300 (N15), normal rabbit IgG, normal mouse IgG, normal goat IgG (SantaCruz Biotech.); anti-Brg1, anti-AcH3, anti-H3K14Ac, anti-H3K27Me (Millipore); anti-Pol II (4H8), anti-Ser5 Pol II, anti-H3, anti-H3K4Me3 (Abcam).

## Supporting Information

Figure S1 *Alad* mRNA levels in J2e and K1-ERp cells. Relative mRNA levels of *Alad* in the K1-ERp parental J2e cell line and K1-ERp cells treated with ethanol or 4-OHT, as determined by Q-RT-PCR. Represented mRNA levels were corrected to *Hprt* mRNA levels. RNA was isolated at 6 h after treatment. *p value≤0.05 by Student’s *t*-test. Data shown represents the average of at least 3 independent experiments (mean ± Std dev).
(PDF)

Figure S2 *p18 Ink4c* RNA levels in K562 cells. Relative mRNA levels of *p18 Ink4c* in K562 cells treated with Hemin to induce terminal differentiation (positive control) and transfected with vector plasmid or KLF1 expression plasmid, as determined by Q-RT-PCR. Represented mRNA levels were corrected to *Hprt* mRNA levels and relative expression determined by the ΔΔCt method. RNA was isolated at 48 h after treatment or transfection. *p value≤0.05 by Student’s *t*-test. Data shown represents the average of at least 3 independent experiments (mean ± Std dev).
(PDF)

Figure S3 *Gata-1*, *Ldb1* and *p45Nf-e2* mRNA levels in K1-ERp cells. Relative mRNA levels of *Gata-1*, *Ldb1* and *p45Nf-e2* in K1-ERp cells treated with ethanol or 4-OHT, as determined by semi-quantitative real-time PCR. Represented mRNA levels were corrected to *Hprt* mRNA levels. RNA was isolated 6 h after treatment. *p value≤0.05 by Student’s *t*-test. Data shown represents the average of at least 3 independent experiments (mean ± Std dev).
(PDF)

Figure S4 KLF1 enhances the deposition of histone modification associated with active genes. ChIP performed on chromatin derived from K1-ERp cells treated with either vehicle control or 4-OHT. Antibodies were specific against (A) histone H3K4Me3, (B) acetylated histone H3. Target DNA enrichment relative to input was determined by Q-PCR using *Alad1b* promoter primers. *p value≤0.05. **p value ≤0.01 by Student’s *t*-test. Data shown represents the average of at least 3 independent experiments (mean ± SEM).
(PDF)
