# Supplementary figures and images for: Regulation of Delta-Aminolevulinic Acid Dehydratase by Krüppel-Like Factor 1
Source: PLoS One. 2012 Oct 3;7(10):e46482. doi: 10.1371/journal.pone.0046482 (PMC3463598; doi:10.1371/journal.pone.0046482)

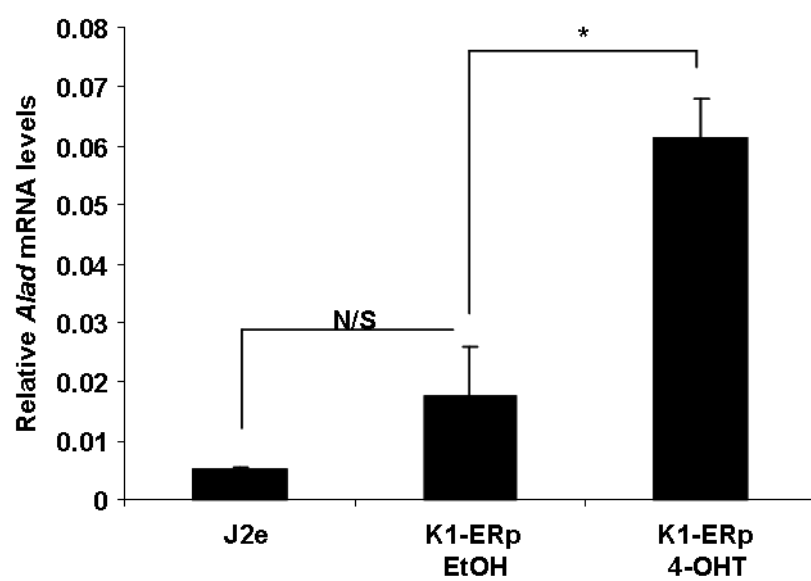

Supplement: Figure S1 — Alad mRNA levels in J2e and K1-ERp cells. Relative mRNA levels of Alad in the K1-ERp parental J2e cell line and K1-ERp cells treated with ethanol or 4-OHT, as determined by Q-RT-PCR. Represented mRNA levels were corrected to Hprt mRNA levels. RNA was isolated at 6 h after treatment. *p value≤0.05 by Student’s t-test. Data shown represents the average of at least 3 independent experiments (mean ± Std dev). (PDF) [file pone.0046482.s001.pdf]

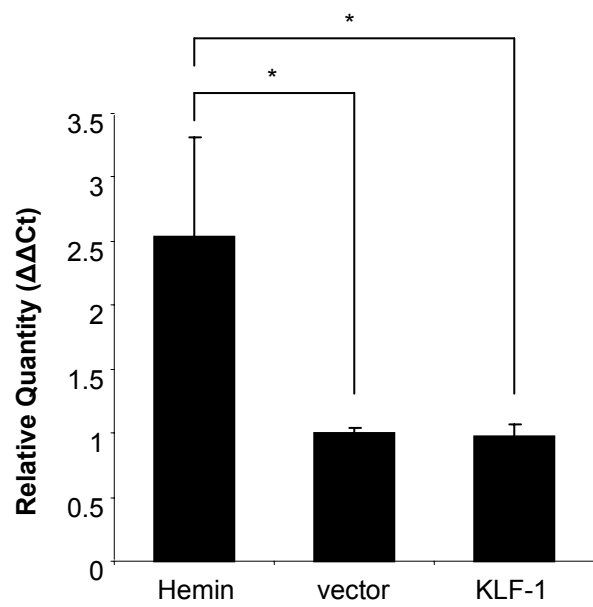

Supplement: Figure S2 — p18 Ink4c RNA levels in K562 cells. Relative mRNA levels of p18 Ink4c in K562 cells treated with Hemin to induce terminal differentiation (positive control) and transfected with vector plasmid or KLF1 expression plasmid, as determined by Q-RT-PCR. Represented mRNA levels were corrected to Hprt mRNA levels and relative expression determined by the ΔΔCt method. RNA was isolated at 48 h after treatment or transfection. *p value≤0.05 by Student’s t-test. Data shown represents the average of at least 3 independent experiments (mean ± Std dev). (PDF) [file pone.0046482.s002.pdf]

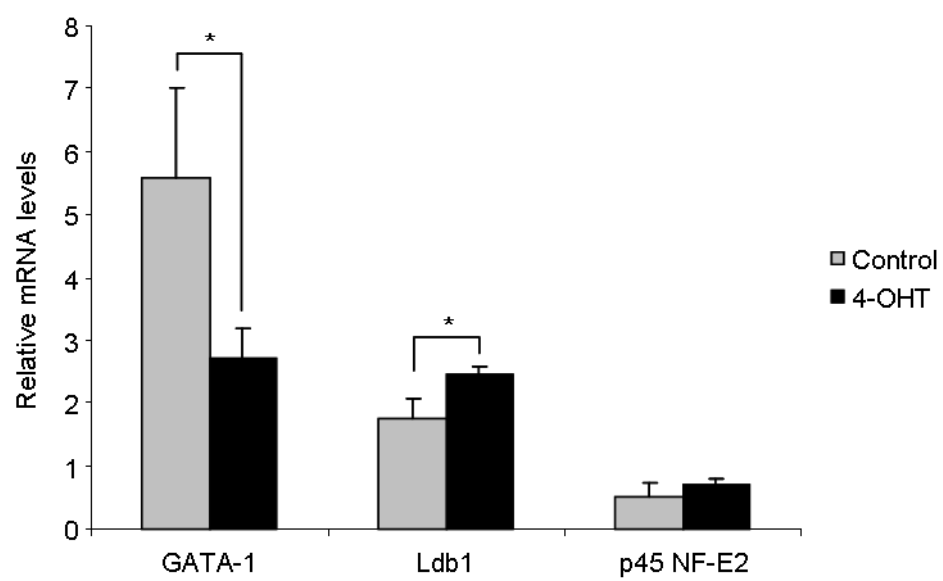

Supplement: Figure S3 — Gata-1, Ldb1 and p45Nf-e2 mRNA levels in K1-ERp cells. Relative mRNA levels of Gata-1, Ldb1 and p45Nf-e2 in K1-ERp cells treated with ethanol or 4-OHT, as determined by semi-quantitative real-time PCR. Represented mRNA levels were corrected to Hprt mRNA levels. RNA was isolated 6 h after treatment. *p value≤0.05 by Student’s t-test. Data shown represents the average of at least 3 independent experiments (mean ± Std dev). (PDF) [file pone.0046482.s003.pdf]

**A**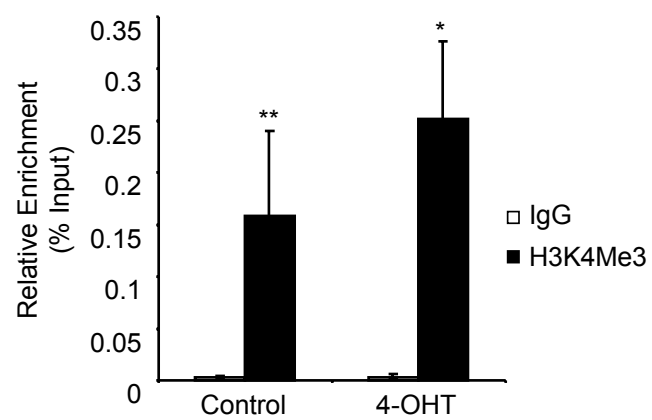**B**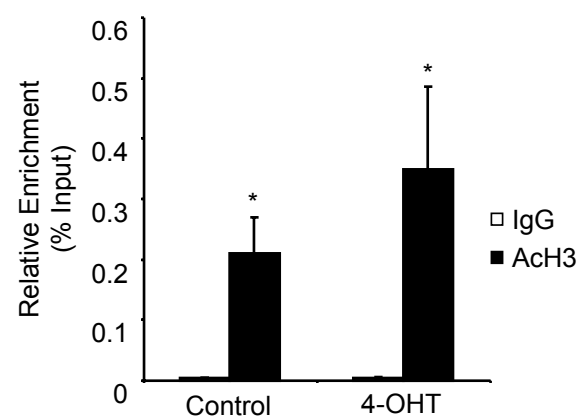

Supplement: Figure S4 — KLF1 enhances the deposition of histone modification associated with active genes. ChIP performed on chromatin derived from K1-ERp cells treated with either vehicle control or 4-OHT. Antibodies were specific against (A) histone H3K4Me3, (B) acetylated histone H3. Target DNA enrichment relative to input was determined by Q-PCR using Alad1b promoter primers. *p value≤0.05. **p value ≤0.01 by Student’s t-test. Data shown represents the average of at least 3 independent experiments (mean ± SEM). (PDF) [file pone.0046482.s004.pdf]
